# Opening up Peer Review in *Life*: Towards a Transparent and Reliable Process

**DOI:** 10.3390/life4020225

**Published:** 2014-05-16

**Authors:** Pabulo Henrique Rampelotto

**Affiliations:** Interdisciplinary Center for Biotechnology Research, Federal University of Pampa, Antônio Trilha Avenue, P.O. Box 1847, 97300-000, São Gabriel–RS, Brazil; E-Mail: pabulo@lacesm.ufsm.br

Peer review is one of the oldest and most respected instruments of quality control in science and it is at the heart of academic publishing. However, while publishing has evolved and adapted to the digital era, peer review remains stuck in its traditional format. The traditional model of peer review relies on undisclosed reviews in which the whole process remains hidden from the scientific community. Despite its importance, the current system lacks sufficient quality and transparency, and has been repeatedly criticized as prone to bias or even fraud. In addition, the traditional peer review process opposes the desired open collaboration for the benefit of science and society.

In view of the fundamental role of peer review in the publication of research and the growth of scientific knowledge, it is essential to develop innovative ways to improve the process. Changes to the system are necessary and the open access movement provides a context to radically reform scientific publishing. During the last decade, open access journals have altered the scientific publishing landscape, making it cheaper and easier for researchers to access published articles. Nowadays, it is high quality open access journals that are leading the development of new approaches to peer review.

As an advocate of transparency in the peer review process, during the last months I have been working with MDPI to implant a new system of open peer review, under which the peer-review reports and authors’ responses are published as an integral part of the final version of each article. This new model of publishing associated with the open access platform of MDPI result in one of the most transparent, unbiased, democratic and reliable assessment of research currently available. The move towards an open peer-review policy gives credit where it is due, but moreover provides valuable information to those reading the article, sharing the reviewers’ critiques of the manuscript and presenting all the necessary information for them to make an objective evaluation for themselves. Furthermore, the available information also benefits young students and scientists who can learn about the reviewing process of a manuscript, which will in turn help them to prepare high quality papers. 

Open review is an established part of the evaluation process for some prominent open access journals such as the BMC-series medical journals, EMBO journals and eLife. Now, *Life* is the first MDPI journal to make this courageous step towards open peer-review in order to demonstrate the rigorous, fair and efficient standard of the editorial work. The first paper published under this new policy was a manuscript written by a Nobelist and reviewed by three experts in the field (available at [1]). 

This transparent process will help to eradicate potential flaws associated with the traditional peer review route. As a result of this unique system, all reviewers will get their due recognition and respect once their names are published with the papers. If reviewers do not want to reveal their identities, we will honour that request and the reviewer’s report will be published “anonymously”. In an initial test phase, authors submitting manuscripts to *Life* will therefore have the choice (after acceptance of their paper) to publish the reviewers’ comments and their responses along with the article. To protect the impartiality of the peer-review process, the identity of the reviewers will not be revealed to the authors until after a paper is accepted for publication.

As highlighted in my previous editorial [2], this is an exciting moment for *Life* and I invite you to submit your manuscript and enjoy a pleasant experience while working with our editorial staff.
